# The All-on-4 Concept Using Polyetheretherketone (PEEK)—Acrylic Resin Prostheses: Follow-Up Results of the Development Group at 5 Years and the Routine Group at One Year

**DOI:** 10.3390/biomedicines11113013

**Published:** 2023-11-09

**Authors:** Miguel de Araújo Nobre, Carlos Moura Guedes, Ricardo Almeida, António Silva, Nuno Sereno

**Affiliations:** 1 Research, Development and Education Department, MALO CLINIC, Avenida dos Combatentes, 43, Level 11, 1600-042 Lisboa, Portugal; 2 Research, Prosthodontic Department, MALO CLINIC, Avenida dos Combatentes, 43, Level 10, 1600-042 Lisboa, Portugal; cguedes@maloclinics.com (C.M.G.); ralmeida@maloclinics.com (R.A.); 3 MALO CLINIC Ceramics, Avenida dos Combatentes, 43, Level 11, 1600-042 Lisboa, Portugal; amsilva@maloceramics.com; 4 Invibio Biomaterial Solutions & JUVORA, Global Technology Center, Hillhouse International, Thornton, Cleveleys FY5 4QD, UK; nsereno@invibio.com

**Keywords:** dental implants, immediate dental implant loading, polyetheretherketone, PEEK, prostheses, implants

## Abstract

Background: It is necessary to investigate the application of polymer materials in implant dentistry. The aim of this study was to examine the outcome of full-arch polyetheretherketone (PEEK)—acrylic resin implant-supported prostheses. Methods: Seventy-six patients were rehabilitated consecutively with 100 full-arch implant-supported prostheses of PEEK–acrylic resin (a development group (DG): 37 patients with 5 years of follow-up; a routine group (RG): 39 patients with 1 year of follow-up). The primary outcome measure was prosthetic survival. Secondary outcome measures were implant survival, marginal bone loss, biological complications, prosthetic complications, veneer adhesion, plaque levels, bleeding levels, and a patient subjective evaluation (including the Oral Health Impact Profile for the RG). Results: In both groups, prosthetic (DG: 93.6%; RG: 100%) and implant survival (DG: 98.9%; RG: 99.5%) were high, and marginal bone loss was low (DG: 0.54 mm; RG: 0.28 mm). The veneer adhesion rate was 28.6% of prostheses in DG (RG = 0%). Mechanical complications occurred in 49% and 11.8% of prostheses in DG and RG, respectively. Biological complications, plaque, and bleeding levels were low in both groups. The subjective patient evaluation was excellent in both groups (8.6 < DG < 8.8; 9.3 < RG < 9.5; OHIP = 1.38). Conclusions: Within the limitations of this study, PEEK can be considered a viable prosthetic alternative.

## 1. Introduction

The use of alternative materials for implant-supported rehabilitations is the object of constant development. One such alternative is polyetheretherketone (PEEK), a high-performance thermoplastic polymer whose application was extended from the aerospace and automotive industry [1] to medicine [2,3,4] and dentistry [5,6,7,8,9,10,11,12], either alone or reinforced with polymer composite materials [13,14]. Given its original development (Victrex plc, Lancashire, UK), the properties that make PEEK an exciting alternative include creep and wear resistance, biostability, biocompatibility, superior mechanical behavior, and compatibility with medical diagnostic imaging [15].

Its use in implant dentistry has received a significant amount of attention, from the scientific community over the last decade from implants [16] to abutments [17] to infrastructures in full-arch implant-supported rehabilitations [6,7,8,9,10,11,18,19,20,21,22,23]. Regarding the latter, the use of PEEK has been involved in some disagreement over its potential favorable or detrimental effects on bone and dental implants, with opposing conclusions from finite element analysis studies [24,25,26,27,28,29]. Concurrently, short-term clinical studies register beneficial effects for full-arch rehabilitations, particularly on marginal bone loss, where important reductions have been noted [6,9,11]. Previous studies registered positive outcomes at 1 and 3 years for PEEK—acrylic resin prosthesis—when applied to full-arch implant-supported rehabilitations, specifically the All-on-4 concept (Nobel Biocare AB, Gothenburg, Sweden) [6,9]. Nevertheless, its effects in the long term and the impact on the patient’s quality of life are lacking.

A second point of debate in the use of PEEK concerns the potential difficulty of the adhesion of veneering materials (either due to PEEK’s properties or the need to follow strict protocols). This renders a significant number of complications previously reported [6,9] that impact the satisfaction of both patient and clinician due to the burdensome caused by increased visits to resolve the complication. This highlights the need to establish a proper preventive protocol.

The aim of this study was to document the outcome of full-arch implant-supported fixed hybrid PEEK—acrylic resin prosthesis—applied to the All-on-4 concept at 5 years for a development group and at 1 year for a routine group with an updated protocol to prevent veneer adhesion issues.

## 2. Materials and Methods

This prospective cohort clinical study was performed in a private practice (Lisbon, Portugal) for a duration of 5 years. This study was approved by an independent ethical committee (Ethical Committee for Health, Lisbon, Portugal; authorization no. 008/2013). Written informed consent was obtained from all patients. Data were divided into two groups as follows: the development and routine groups. The patients were assigned consecutively to a development group and were rehabilitated between May 2015 and October 2016, and a routine group was rehabilitated between November 2017 and April 2021.

Seventy-six patients (53 females, 23 males), with an average of 58.5 years (range: 20–80 years, standard error of the mean: 1.23 years), were rehabilitated with 100 full-arch implant-supported prostheses (27 maxillary rehabilitations, 25 mandibular rehabilitations, and 24 bimaxillary rehabilitations). The development group included 37 patients (29 females, 7 males), with an average age of 59.8 years (standard error of the mean: 1.77 years), who were rehabilitated with 49 full-arch prostheses (12 maxillary rehabilitations, 13 mandibular rehabilitations, and 12 bimaxilar rehabilitations). The routine group included 39 patients (24 females, 15 males) with an average age of 57.1 years (standard error of the mean: 1.70 years), who were rehabilitated with 51 full-arch prostheses (15 maxillary rehabilitations, 12 mandibular rehabilitations, and 13 bimaxilar rehabilitations).

### 2.1. Inclusion and Exclusion Criteria

The patients who met the inclusion criteria were identified at the treatment planning phase. Inclusion criteria consisted of patients with full-arch rehabilitations (single arch or bimaxillary rehabilitations) performed using the All-on-4 concept (Nobel Biocare AB, Gothenburg, Sweden), in need of definitive prosthetic rehabilitation, and who provided written informed consent to participate. Exclusion criteria included patients unable to provide written informed consent, with insufficient bone volume, or inactive radiotherapy or chemotherapy.

### 2.2. Surgical and Prosthetic Protocols

Implant insertion (Nobelspeedy^TM^, Nobel Biocare AB, Gothenburg, Sweden) followed standard procedures [30] except for the use of under-preparation, which was employed to guarantee a final torque of over 32 N/cm before the final implant seating. The two most anterior implants were inserted following the direction determined by the anatomy of the jaw. Two posterior implants were inserted (one implant on each quadrant) anterior to the mental foramina (in the mandible) and the anterior wall of the maxillary sinus (in the maxilla) with distal tilting between 30 and 45 degrees relative to the occlusal plane *ad modum* All-on-4 concept (Nobel Biocare AB).

Concerning the immediate prosthetic protocol, a high-density acrylic resin (PalaXpress Ultra; Heraeus Kulzer GmbH, Hanau, Germany) prostheses with titanium cylinders (Nobel Biocare AB, Gothenburg, Sweden), and a minimum of 10 teeth were manufactured at the dental laboratory and inserted on the same day. Anterior occlusal contacts and canine guidance during lateral movements were preferred as the occlusal scheme.

**Figure 1:**
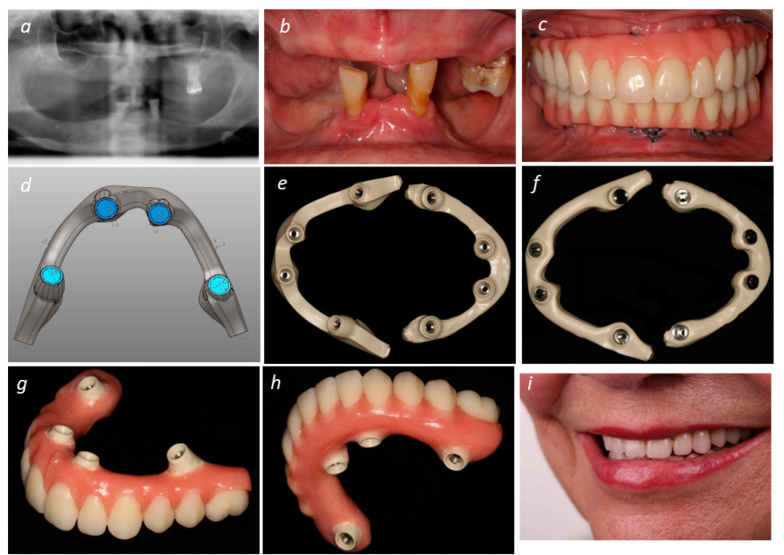

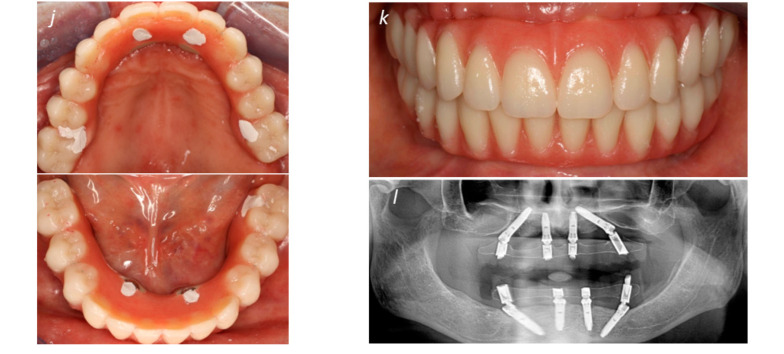
(**a**) Pre-treatment orthopantomography; (**b**) Pre-treatment intraoral view of both arches; (**c**) Immediate provisional prostheses after bimaxilar full-arch rehabilitation; (**d**) Infrastructure design during CAD–CAM process; (**e**) Polyetheretherketone (PEEK) infrastructures inferior view during CAD–CAM process; (**f**) PEEK infrastructures with a superior view during CAD–CAM process; (**g**) Maxillary PEEK–acrylic resin implant-supported prosthesis; (**h**) Mandibular PEEK–acrylic resin implant-supported prosthesis; (**i**) Perspective of the patient’s smile after the rehabilitation process; (**j**) Intra-oral occlusal view of the maxillary and mandibular PEEK–acrylic resin implant-supported prostheses in function; (**k**) Intra-oral frontal view of the maxillary and mandibular PEEK–acrylic resin implant-supported prostheses in function; (**l**) Final post-treatment orthopantomography.

The definitive prosthetic protocol was described in full-on previous publications [6,9]. In brief, it consisted of a full-arch hybrid prostheses of polymeric–acrylic resin implant-supported and fixed prostheses (patent WO 2019/008368) [31] with a PEEK substructure (Juvora Ltd., Lancashire, UK), reinforcing titanium sleeves, acrylic resin prosthetic teeth (anterior teeth: Premium; posterior teeth: Mondial; Heraeus Kulzer GmbH, Hanau, Germany) and pink acrylic resin gingiva (PalaXpress Ultra, Heraeus Kulzer GmbH, Hanau, Germany). In the development group, the metal bond 1 and 2 (Heraeus Kulzer GmbH, Hanau, Germany) was used as a primer (Signum Connector, Heraeus Kulzer GmbH, Hanau, Germany) in the cases identified with veneer adhesion issues; in the routine group, the Signum connector (Heraeus Kulzer GmbH, Hanau, Germany) was used on all cases. The infrastructure CAD–CAM guidelines included an “I” shaped design in the framework, with minimum cross-sectional material dimensions of 5 mm of occlusal–cervical height, 4 mm of anterior buccal–lingual width, and 6 mm of buccal–lingual width in the areas of the titanium sleeve, together with at least 1 to 2 mm of acrylic resin. The final prostheses privileged a mutually protected occlusion scheme respecting the patients’ centric relations. A clinical case is represented in Figure 1.

### 2.3. Maintenance Protocol

The connection of the definitive prosthesis was considered the baseline for clinical and radiographic evaluations, with the patients included in a maintenance protocol with clinical evaluations every 6 months and both clinical and radiographical evaluations at 1 and 5 years.

### 2.4. Outcome Measures

The primary outcome measure at five years was prosthetic survival (the need for replacement), including the fracture of the framework. Secondary outcome measures were implant survival, marginal bone loss, technical evaluation concerning manufacturing issues, the incidence of mechanical complications, the incidence of biological complications, modified plaque index (mPLI), modified bleeding index (mBI), and patient subjective evaluation. Implant survival was evaluated based on function and using the patient as a unit of analysis (with the first implant failure in a patient considered as a censoring event irrespective of the remaining implants maintaining their function) [6]. Marginal bone loss was evaluated through periapical radiographs employing a radiographic holder (super-bite; Hawe Neos, Bioggio, Switzerland), adjusted for the digital film’s orthognathic position. The radiographs were evaluated using an outcome assessor through software for image analysis (rayMage, version 2.3, MyRay, Imola, Italy). The marginal bone level was defined as the distance between the implant’s platform and the most apical bone–implant contact, while the measurement difference between the baseline (connection of the definitive prosthesis) and five-year evaluation was classified as marginal bone loss (MBL). We calibrated the measurements using the distance between implant threads and considered average values between the mesial and distal sites. The technical evaluation concerning manufacture issues was evaluated comprising the following: infrastructure manufacture issues (presence or absence), framework integrity issues (present/absent), and veneer adhesion issues (present or absent). The biological complications assessed were as follows: a probing pocket depth >4 mm, evaluated using a plastic periodontal probe calibrated to 0.25 N; abscess (presence or absence); fistulae formation (presence or absence); suppuration (presence or absence); and patient adverse soft tissue reaction (presence or absence). The mechanical complications assessed were the loosening or fracture of prosthetic screws, abutments, or prosthesis. The modified plaque index (mPLI) [32] was evaluated by inserting a periodontal plastic probe 1 mm into the peri-implant sulcus, running a circular movement all around the implant, and measuring in a scale between 0 and 3 (0, no detection of plaque; 1, plaque only recognized by running a probe across the smooth marginal surface of the implant; 2, plaque can be seen by the naked eye; and 3, abundance of soft matter). The modified bleeding index (mBI) [32] was evaluated on the same moment as mPLI and measured on a scale between 0 and 3 (0, no bleeding when a periodontal probe is passed along the mucosal margin adjacent to the implant; 1, isolated bleeding spots visible; 2, blood forms a confluent red line on mucosal margin; and 3, heavy or profuse bleeding). The patients’ evaluation comprised the “in mouth comfort” defined as the following: the comfort felt by the patient with the prosthesis in function regarding an overall fulfillment of expectations, measured in a visual analog scale between 0 (poor) and 10 (excellent), and the “overall chewing feeling”, defined as the patients’ feeling during daily food intake routines in relation to their ability to chew any type of food and measured in a visual analog scale between 0 (poor) and 10 (excellent). Both evaluations were registered yearly. For the routine group, an additional evaluation was performed using the Oral Health Impact Profile, version 14 (OHIP-14) [33], estimating the following dimensions: functional limitations, physical pain, psychological discomfort, physical disability, psychological disability, social disability, and handicap; this was measured using a Likert scale between 0 (never occurred) and 4 (always occurs) at 6 months and 1 year of follow-up.

### 2.5. Statistical Analysis

Survival was estimated using life table analysis (actuarial method) and using the implant and prosthesis as a unit of analysis. Descriptive statistics (average, standard deviation) were computed for the following variables of interest: age, marginal bone loss and patient evaluation parameters, OHIP-14 dimensions, “in mouth comfort”, and “overall chewing feeling”. The median was computed for the variables mPLI and mBI; frequencies were computed for the technical evaluation concerning manufacturing issues, including the incidence of biological and mechanical complications. Inferential analysis was computed for the evaluation of the correlation between mPLI and mBI through the Spearman correlation coefficient. The significance level was set at 5%. Data were analyzed using the software SPSS for Windows (IBM SPSS, New York, NY, USA) version 17.

## 3. Results

### 3.1. Sample

For the development group, the median cantilever length in a prosthesis was 1 unit (average: 0.7; standard deviation: 0.6 units; range: 0–2 units). Two female patients (5.4%) with two single full-arch maxillary prostheses (4.1%) were lost to follow-up as they became inaccessible; one female patient (2.7%) with a single full-arch mandibular prosthesis (2%) deceased due to cancer after 39 months; and one female patient (2.7%) with a single full-arch mandibular prosthesis (2.0%) withdrew from the study after 48 months. A total of 33 patients (89.2%) with 45 prostheses (91.8%) were eligible for follow-up and completion at 5 years.

For the routine group, the median cantilever length in a prosthesis was 1 unit (average: 0.55; standard deviation: 0.57 units; range: 0–2 units). Two patients (5.1%) with two double full-arch prostheses (7.8%) were lost to follow-up/withdrew during the first year of follow-up: one patient deceased due to health conditions unrelated to the prosthodontic treatment, and one patient chose to be followed at another dental clinic. A total of 37 patients (94.9%) with 47 prostheses (92.2%) were evaluated at 1 year of follow-up.

### 3.2. Primary Outcome Measure—Prosthetic Survival

For the development group, three PEEK frameworks were fractured as follows: one mandibular framework in bimaxilar rehabilitation during the first year of follow-up in a male patient, heavy bruxer, 55 years of age requiring a new prosthesis; one mandibular framework was characterized by the complete fracture of the mandibular PEEK framework near the cylinder (position #42) during the fourth year of follow-up in a female patient 75 years of age that developed bruxing habits (and deceased due to cancer shortly after); one maxillary framework fractured on the cantilever position (position #26) in a male patient at 47 years of age (that refused its replacement as the prosthesis remained in function). This outcome rendered a 93.6% prosthetic cumulative survival rate at five years of follow-up (Table 1).

No prosthesis failed in the routine group during the first year of follow-up, resulting in a 100% prosthesis survival rate (Table 1).

### 3.3. Secondary Outcome Measures

#### 3.3.1. Implant Survival

A total of 196 implants were inserted for the rehabilitation of 49 edentulous arches in the development group. Two implant failures (implant positions #32 and #34) were registered in one female patient 75 years of age (the same patient deceased due to cancer) during the fourth year of follow-up, rendering a 98.9% implant survival rate after five years (Table 2).

Concerning the routine group, a total of 204 implants were inserted for the rehabilitation of 51 edentulous arches. One implant failure was registered in one female patient with mandibular rehabilitation (implant position: #32) at 7 months of follow-up, with increased marginal bone loss noted during the rehabilitation process, rendering a 99.5% implant survival rate after one year (Table 2).

#### 3.3.2. Marginal Bone Loss

For the development group, the average (standard deviation) marginal bone loss at 5 years was 0.54 mm (0.95 mm) with the following distribution: 0.42 mm (0.95 mm) for maxillary implants; 0.65 mm (0.94 mm) for mandibular implants; 0.42 mm (0.89 mm) for bimaxillary rehabilitations; 0.78 mm (0.99 mm) for single arch maxillary rehabilitations; and 0.62 mm (1.03 mm) for single arch mandibular rehabilitations. Concerning the routine group, the average (standard deviation) marginal bone loss at one year of follow-up was 0.28 mm (0.59 mm), with 0.25 mm (0.54 mm) for maxillary implants; 0.32 (0.65 mm) for mandibular implants; 0.27 mm (0.55 mm) for bimaxillary rehabilitations; 0.22 mm (0.48 mm) for single arch maxillary rehabilitations; and 0.37 mm (0.76 mm) for single arch mandibular rehabilitations.

#### 3.3.3. Technical Evaluation—Veneer Adhesion Issues

A total of 12 patients (32.4%) and 14 prostheses (28.6%) registered veneer adhesion issues in the development group during the 5 years of follow-up (Table 3). These were characterized by the avulsion of acrylic resin from the PEEK infrastructure. All situations were solved by leaving the cylinder areas with increased amounts of exposed PEEK to increase flexion resistance; a tungsten bur was used to increase mechanical retention on the PEEK infrastructure and the bonding primer was replaced to increase the tensile bond strength. On the routine group, no veneer adhesion issues were registered during the first year of follow-up.

#### 3.3.4. Mechanical Complications

The incidence of mechanical complications on the development group was 54.1% at the patient level (n = 20 patients) and 49% at the prostheses level (n = 24 prostheses). In the routine group, the incidence rate of mechanical complications was 12.8% at the patient level (n = 5 patients) and 11.8% at the prostheses level (n = 6 prostheses). Table 4 describes the type of complications and remedies implemented, with all situations resolved in both groups.

#### 3.3.5. Biological Complications

The incidence rate of biological complications during the 5 years of follow-up for the development group was 10.8% at the patient level (n = 4 patients) and 2.6% at the implant level (n = 5 implants) consisting of a peri-implant pathology. Table 5 describes the type of complications and remedies implemented, with all situations resolved apart from two implants in two patients. No biological complications were registered for the routine group during the follow-up of 1 year.

#### 3.3.6. Plaque and Bleeding Scores

Considering the development group, the median value for the mPLI was 2 (plaque visible by the naked eye) at five years of follow-up; while the mBI median value obtained at five years of follow-up was 1 (one isolated bleeding spot visible when tested). The correlation between plaque and bleeding levels was weak (positive) and non-significant (R = 0.240, *p* = 0.210; Spearman).

Regarding the routine group, the median for the mPLI was 1 (plaque was only recognized by running a probe across the smooth marginal surface of the peri-implant sulcus) at both six months and one year of follow-up. The median for the mBI was 1 (one isolated bleeding spot visible when tested) at both 6 months and one year. The correlation between mPLI and mBI was characterized by a strong positive linear relationship at both 6 months and 1 year (six months: R = 0.609, *p* < 0.001; one year: R = 0.672, *p* < 0.001; Spearman correlation coefficient).

#### 3.3.7. Patient Subjective Evaluation and OHIP-14 Assessment

In the development group at 5 years, “in mouth comfort” registered a mean value of 8.8, while ”overall chewing feeling” registered a mean value of 8.6 and both indexes were evaluated on a scale of 0 to 10 (0, poor; 10, excellent).

**Figure 2:**
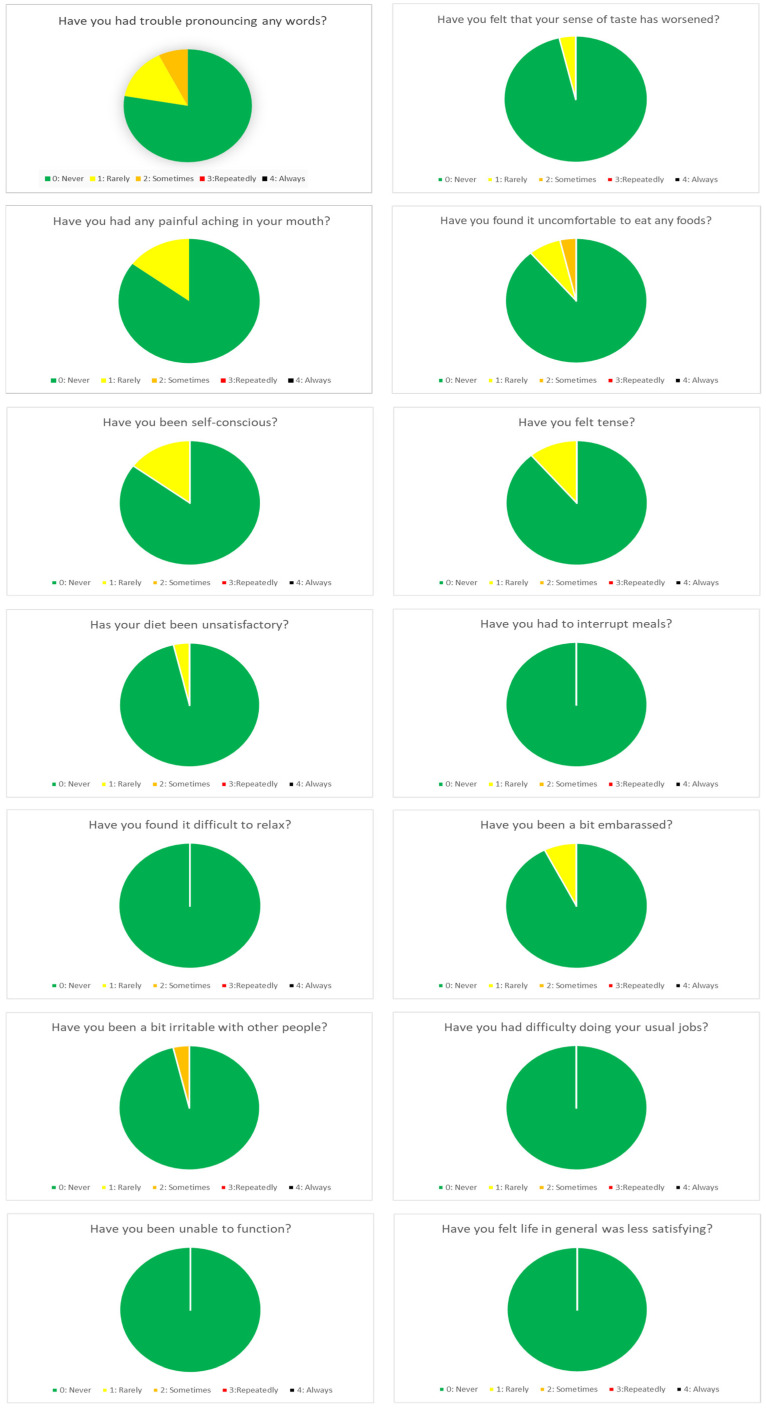
OHIP-14 dimensions at 6 months of follow-up.

**Figure 3:**
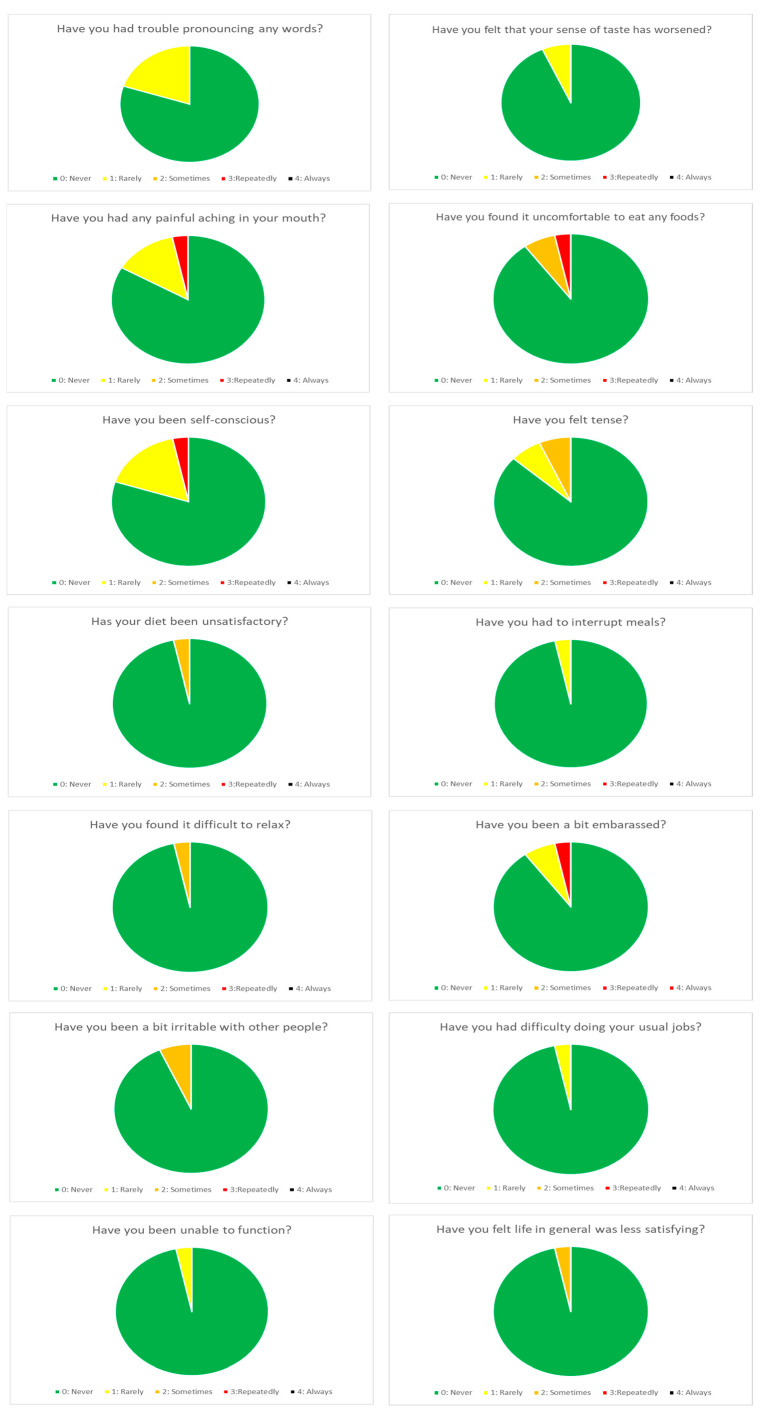
OHIP-14 dimensions at 1 year of follow-up.

In the routine group, the registered mean value for “in mouth comfort” was 9.5 and 9.3 at 6 months and 1-year, respectively, whereas “overall chewing feeling” registered a mean value of 9.3 at 6 months and 9.4 at one year. In addition, concerning the patients’ quality of life evaluation in the OHIP-14 dimensions, the mean total sum OHIP-14-dimension scores were 0.73 and 1.38 at 6 months and 1 year, respectively (Table 6). The distribution was skewed, with scores of 0 reported by 63% of the patients at 6 months and 60% of the patients at 1 year of follow-up (Figure 2 and Figure 3).

## 4. Discussion

The current study reported on the outcome of a full-arch prosthodontic solution consisting of a fixed hybrid implant-supported PEEK–PMMA prosthesis produced through CAD–CAM workflow at 1 and 5 years for two different groups (routine and development, respectively).

The 100% prosthesis survival at one year registered for the routine group and the 93.6% prosthesis survival at five years for the development group are suitable taking into consideration the complexity of full-arch implant-supported rehabilitation and the broad inclusion criteria applied to this study. The PEEK infrastructure fractures corresponded to patients that were heavy bruxers and/or whose structures had >1 cantilever unit, which is a pattern that was also noted for the high incidence of mechanical complications. Bruxism and cantilever units represent two factors that significantly increase the probability of mechanical complications, with a previous study evaluating different implant distributions registering odds ratios >60 times for bruxism and >4.5 times for cantilevers [34]. Nevertheless, it is important to underline the previously mentioned limits established in CAD–CAM guidelines for full-arch PEEK infrastructures together with the limitation to one cantilever unit (≤10 mm) as these were created to compensate for material flexion [6]. Furthermore, the change in the bonding primer for the routine group was beneficial to the protocol considering the excellent results obtained (absence of veneer adhesion issues) together with the successful resolution on the development group’s incident cases. The bonding primer of choice was characterized by a higher tensile bond strength enabling firmer chemical retention [35,36]. In addition, actions were taken to improve mechanical retention, including an increased amount of exposed PEEK to the cylinder areas, a PEEK rough finish [37,38,39], insertion of a horizontal thread in the infrastructure without smooth and round finish, and the insertion of vertical threads in the cantilever area, enabling PEEK’s flexion capacity to be maintained under control. PEEK is a completely different material compared to titanium regarding its mechanical characteristics, so one should approach the prosthetic design accordingly to these features. In circumstances where the space is reduced and the infrastructure does not possess an appropriate height, the shock-absorbing property of PEEK, which is an advantage for dissipating occlusal forces, makes the infrastructure too flexible. In this situation, a titanium infrastructure designed in traditional fashion could be functional. However, in the case of a PEEK infrastructure (37 times more flexible than zirconia) [38], there is too much bending of the cantilevers and pontic areas, causing mechanical complications such as prosthetic screw loosening or fractures and fissures in the pink acrylic areas [40]. Extending the PEEK framework to the gingival tissue and creating areas in the lingual aspect that remain uncovered with acrylic resin reinforces the infrastructure and prevents or minimizes complications.

Implant survival was high (99.5% at 1-year for the routine group; 98.9% at 5 years for the development group) and compared favorably with previous publications on the outcome of full-arch rehabilitations. Recent publications on full-arch implant supported rehabilitations [41,42,43], including two studies on the All-on-4 concept (Nobel Biocare AB) with a long-term outcome [42,43] registered for cumulative survival rates between 98.6% and 99.6% at 1 year and between 97.7% and 98.8% at the 5 years evaluation. A recent systematic review and meta-analysis evaluating the outcome of full-arch rehabilitations supported by tilted and axially placed implants reported an implant survival proportion at 5 years of 98.2% (95% confidence intervals: 97.93%, 98.43%) [41].

The marginal bone loss registered in both groups was low with 0.28 mm (routine group at 1 year) and 0.54 mm (development group at 5 years). These results compare favorably with a recent systematic review and meta-analysis, which was lower than the overall or individual average of all included studies at 1 year or 5 years [39]. In addition, the routine group marginal bone loss at 1 year was lower (−0.09 mm) compared to the development group at the same time frame [6]. The incidence of biological complications was also low during the follow-up of this study (absence in the routine group; 2.6% of the implants in the development group), which is in line with the results from a systematic review (data extracted from study) [41]. All four patients with biological complications presented history of periodontitis or smoking habits, representing risk indicators for the incidence of biological complications [44,45]. The good results registered for these outcome measures (implant survival, marginal bone loss and biological complications) can be potentially attributable to PEEK’s properties, specifically its shock absorption characteristics [9,10,46,47,48]. Recent finite element analysis studies reported conflicting results when evaluating PEEK infrastructures used in full-arch implant-supported rehabilitations and the *ad modum* All-on-4 concept: Tribst et al. [24], Ersöz et al. [26], Yu et al. [27] and Dayan et al. [28] reported that PEEK frameworks transferred more stress to the bone in both compression and tensile stress compared to other metallic frameworks (including titanium) in what could be perceived as placing the rehabilitations at an increased risk of biological complications. Shash et al. [25] and Haroun et al. [29] registered that PEEK material reduced the stresses and strains on bone tissue compared to titanium, and therefore, could prevent complications. In light of these results, with high implant survival and low marginal bone loss, the present study is in agreement with Shash et al. [25] and Haroun et al. [29], a result that is parallel with other clinical investigations [11,20,49]. A clinical study comparing PEEK and titanium frameworks in full-arch implant-supported fixed prosthesis with an average follow-up of 26.5 months registered a significantly lower marginal bone loss for PEEK (0.70 mm) compared to titanium (0.96 mm) [11]. A systematic review investigating the clinical performance of polymer frameworks in dental prostheses registered in their qualitative analysis lower plaque and gingival indices, probing depth, and marginal bone loss, with higher survival rates for implant-supported and fixed prostheses and overdentures fabricated with PEEK than for metal frameworks [20]. Despite the increased number of finite element analysis studies recently published on the subject [24,25,26,27,28,29], it should be noted that they do not mimic the true clinical scenario. This is due to the restrictions of these experiments and an array of possible scenarios including different types of implants; modeling only a portion of bone considered as isotropic material despite its anisotropic behavior; assuming in most cases complete osseointegration; considering compressive or oblique forces acting on the implant; or neglecting muscle forces and the bone remodeling process, thus attesting the absence of a standardized approach for finite element analysis modeling in dentistry [50].

Concerning plaque and bleeding scores, both groups were characterized by the presence of plaque around the implants, with increased plaque levels at 5 years for the development group (visible plaque) compared to a median lower score at 1 year for the routine group (plaque only visible after running the periodontal probe across the mucosal margin). On the other hand, bleeding levels were stable with the median representing mild inflammation for the routine group at 1 year and the development group at 5 years. The correlation between plaque and bleeding scores has been pointed out by previous publications, representing the causality between plaque accumulation and peri-implant breakdown [51,52]. The present study points in the same direction, with a positive correlation between plaque and bleeding scores in both groups, despite the non-significant correlation for the development group. This result should nevertheless be analyzed in the context of the risk for biological complications (which remained at a low level), in what can be inferred as the presence of stable biomechanical conditions that allow to mitigate or delay the deleterious effects of plaque accumulation in the process of peri-implant pathology [10,44]. Nevertheless, it is important to stress the importance of frequent maintenance appointments for soft tissue evaluation, prophylaxis and hygienic measures education [10].

The patients’ subjective evaluation was characterized by a high satisfaction rate for the “in mouth comfort” and “overall chewing feeling” evaluation parameters, with over 90% for the routine group at 1 year and over 85% for the development group at 5 years. Both results were substantially higher (5% to 14%) than the satisfaction rate registered for the fixed mandibular full-arch implant-supported fixed prostheses of metal–acrylic resin regarding an improvement in the chewing ability and the fulfillment of expectations [53]. Moreover, an excellent impact on the patient’s quality of life was registered by the low OHIP-14 index value at both 6 months and 1 year for the routine group. The significance of this result can be described as a potential gain in quality-adjusted life years, a measure of impact which represents population health by considering the duration and quality of life. A study with 9445 subjects estimated the quality-adjusted life-expectancy loss for dental-related events including missing teeth [54]. The authors attested the substantial burden of dental conditions on quality of life, estimating an impact of approximately between ¼ to 1/3 of major causes of health burden such as diabetes, heart disease, obesity and smoking [54]. The present study, with 60% of the participants registering no impact (a score of 0), and only an average of 1.38 for the sample’s quality of life, creates conditions for a large gain in years of life lived with quality by restoring the patients’ function, aesthetics, and self-esteem. This confirms the results of a recent randomized controlled trial evaluating the treatment outcomes (functional and subjective through OHIP-20) of full-arch fixed hybrid rehabilitations of PEEK with milled crowns of a nano-filled composite, concluding that the treatment significantly improved the masticatory performance, bite force, occlusal pattern, quality of life, and satisfaction [18].

The study limitations include the lack of a non-polymeric control group, the short follow-up of the routine group and the fact that this was a single-center study. The strengths of this study include the low rate of dropouts (11% and 5% for the development and routine groups, respectively) which relates to an increased internal validity and the prospective study design. However, the outcomes should be interpreted with caution as even in low dropout rates, the patients missing control appointments had an increased probability of a deleterious outcome, therefore implying an overestimation of the results. Future studies should aim to assess the outcome at midterm and during the longer term for the routine group to evaluate the clinical and patient-centered impacts of the protocol modifications.

## 5. Conclusions

Within the limitations and considering the results of the present study, it can be concluded that when using PEEK as a framework in fixed prosthetic implant-supported prostheses for full-arch rehabilitations *ad modum*, the All-on-4 concept is an acceptable treatment approach. A high prosthetic/implant survival was registered, together with low biological complication rates, low marginal bone loss, and an excellent impact on the patient’s subjective evaluation/quality of life. It further constitutes a shock-absorbing alternative that provides conditions for a beneficial and stable long-term outcome. The protocol modification resulted in an absence of veneer adhesion issues. The high incidence of mechanical complications implies strict respect for the CAD–CAM design and the number of cantilever units.

## 6. Patents

An international patent resulting from the work reported in this manuscript was issued on 10 January 2019: Silva, A.; Legatheaux, J.; de Araújo Nobre, M.; Guedes, C.M.; Almeida, R.; Maló, P.; Sereno, N. Dental prosthesis. International patent no. WO 2019/008368 A1.

## Figures and Tables

**Table 1:** Cumulative prosthetic survival rate for patients rehabilitated using the All-on-4 treatment concept and a hybrid polyetheretherketone (PEEK)—acrylic resin prostheses—for development and routine groups.

	Development Group				
Time	Total Number of Patients	Total Number of Prostheses	Prosthetic Failures	Lost to Follow-Up	Cumulative Survival Rate (%)
Prosthesis connection—1 year	37	49	1	2	98.0%
1 year–2 years	35	46	0	0	98.0%
2 year–3 years	35	46	0	1	98.0%
3 years–4 years	34	45	1	0	95.8%
4 years–5 years	34	44	1	2	93.6%
	**Routine Group**				
**Time**	**Total Number of Patients**	**Total Number of Prostheses**	**Prosthetic Failures**	**Lost to Follow-Up**	**Cumulative Survival Rate (%)**
Prosthesis connection—1 year	39	51	0	2	100%

**Table 2:** Cumulative implant survival rate for patients rehabilitated using the All-on-4 treatment concept and a hybrid polyetheretherketone (PEEK)—acrylic resin prostheses— for the development and routine groups.

	Development Group			
Time	Total Number of Implants	Implant Failures	Lost to Follow-Up	Cumulative Survival Rate (%)
Prosthesis connection—1 year	196	0	8	100.0%
1 year–2 years	188	0	0	100.0%
2 year–3 years	188	0	0	100.0%
3 years–4 years	184	2	2	98.9%
4 years–5 years	180	0	4	98.9%
	**Routine Group**			
**Time**	**Total Number of Prostheses**	**Prosthetic Failures**	**Lost to Follow-Up**	**Cumulative Survival Rate (%)**
Prosthesis connection—1 year	204	1	8	99.5%

**Table 3:** Veneer adhesion problems between acrylic resin and polyetheretherketone (PEEK) infrastructure and resolution. All situations occurred in the development group.

Patient	Gender	Follow-Up (Months)	Position (FDI)	Type Rehabilitation	Opposing Dentition	Resolution
1	Male	5	#12, #22, #25, #35	Bimaxilar	Implant-supported prosthesis	New prostheses due to fracture of PEEK infrastructure
2	Male	2	#35	Mandibular	Mucosal-retained full-arch prosthesis	To increase flexion resistance, the cylinder areas were left with increased amounts of exposed PEEK; to increase mechanical retention in the PEEK infrastructure, a tungsten bur was used; to increase the tensile bond strength, the bonding primer was replaced
3	Female	4	#46	Mandibular	Natural teeth and implant-supported prosthesis	
4	Female	10	#45	Mandibular	Mucosal-retained full-arch prosthesis	
5	Female	12	#35	Mandibular	Mucosal-retained full-arch prosthesis	
6	Female	12	#15, #22	Bimaxilar	Implant-supported prosthesis	
7	Female	16	#26	Maxillary	Natural teeth	
8	Female	30	#35	Mandibular	Implant-supported prosthesis	
9	Male	32	#12	Maxillary	Implant-supported prosthesis	
10	Female	52	#15	Maxillary	Natural teeth	
11	Male	53	#12, #13, #22, #45, #46	Bimaxilar	Implant-supported prosthesis	
12	Female	55	#13	Bimaxilar	Implant-supported prosthesis	

**Table 4:** Incidence of mechanical complications and resolutions during the 5 years of follow-up of the sample.

Patient	Gender	Opposing Dentition	Cantilever Units (Left/Right)	Follow-Up in Months	Acrylic Resin Crown Fracture (Position FDI)	Abutment Wearing (Position FDI)	Abutment Loosening (Position FDI)	Prosthetic Screw Loosening (Position FDI)	Prosthetic Screw Fracture (Position FDI)	Resolution
DG 1	Male	ISP	0/0 (maxilla); 1/1 (mandible)	5	#12, #22; #35					1; Patient fractured PEEK infrastructure
DG 2	Male	ISP	1.5/0.5	16	#32		#42			1
DG 3	Male	ISP	1.5/2	22	#41					1
DG 4	Female	NT	1/1	15		#45				2
DG 5	Female	ISP	1/0.5	16			#45	#42		3
DG 6	Female	ISP	2/2	16			#42			3
DG 7	Female	ISP	1/1 (maxilla); 0/0 (mandible)	8				#25, #35, #45		3
DG 8	Female	ISP	0/0	4				#15		3
DG 9	Male	NT	1/1	20				#16, #26		3
DG 10	Female	RP	2/2	40	#35					1
DG 11	Female	NT	0/0	43					#12, #26	2
DG 12	Female	ISP	1/0	43			#32			2
DG 13	Female	ISP	1/1	47	#12					1
DG 14	Female	NT	0/0	48	#12					1
DG 15	Male	RP	1/1	49					#35	2
DG 16	Male	ISP	0/0	55			#15			2
DG 17	Female	ISP	1/1	55				#25		3
DG 18	Female	ISP	1/1	59	#11					1
DG 19	Female	ISP	1/1	59	#31					1
DG 20	Female	NT	0/0	60	#31,#41					1
RG 1	Female	RP	2/1	6				#42		3
RG 2	Female	ISP	0/0	6			#12, #22			2
RG 3	Female	ISP	1/1 (maxilla); 2/2 (mandible)	12; 6			#12, #13 #31, #32, #41			1
RG 4	Female	FPNT	1/1	12				#25		3
RG 5	Female	NT	1/1	12			#15, #25		#22	1; 2

DG: development group; RG: routine group; ISP: implant support prosthesis; NT: natural teeth; RP: removable prosthesis; FPNT: fixed prosthesis over natural teeth. Resolutions: 1—mending the prostheses and adjusting occlusion; 2—replacing the abutment/prosthetic screw and adjusting occlusion; 3—torque-controlled retightening and adjusting occlusion.

**Table 5:** Incidence of biological complications (peri-implant pathology) and resolutions during the 5 years of follow-up (all from the development group).

Patient	Gender	Implant Position (FDI)	Presence of Risk Indicators	Time of Follow-Up	Resolution
1	Male	#12	Smoker; History of Periodontitis	48 months	Resolved non-surgically
2	Female	#26	Smoker; History of Periodontitis	52 months	Resolved non-surgically
3	Female	#45	History of Periodontitis	55 months	Not resolved
4	Male	#35	History of Periodontitis	58 months	Not resolved

**Table 6:** OHIP-14 scores and patient’s subjective evaluation of the routine group.

OHIP-14 Evaluation Parameters	6 Months Mean (Standard Error of Mean)	1 Year Mean (Standard Error of Mean)
*Functional limitation*	0.23 (0.06)	0.20 (0.04)
Have you had trouble pronouncing any words?		
Have you felt that your sense of taste has worsened?		
*Physical pain*	0.20 (0.06)	0.35 (0.08)
Have you had painful aching in your mouth?		
Have you found it uncomfortable to eat any foods?		
*Psychological discomfort*	0.18 (0.05)	0.38 (0.09)
Have you been self-conscious?		
Have you felt tense?		
*Physical disability*	0.03 (0.02)	0.08 (0.04)
Has your diet been unsatisfactory?		
Have you had to interrupt meals?		
*Psychological disability*	0.05 (0.03)	0.18 (0.06)
Have you found it difficult to relax?		
Have you been a bit embarrassed?		
*Social disability*	0.05 (0.04)	0.13 (0.05)
Have you been a bit irritable with other people?		
Have you had difficulty doing your usual jobs?		
*Handicap*	0.00 (0.00)	0.08 (0.04)
Have you been unable to function?		
Have you felt life in general was less satisfying?		
*Total sum*	0.73 (0.00)	1.38 (0.00)

## Data Availability

Data are available from the authors upon reasonable request.
